# Combination of GD2-directed bispecific trifunctional antibody therapy with Pd-1 immune checkpoint blockade induces anti-neuroblastoma immunity in a syngeneic mouse model

**DOI:** 10.3389/fimmu.2022.1023206

**Published:** 2023-01-09

**Authors:** Sara Marie Ivasko, Kathleen Anders, Laura Grunewald, Michael Launspach, Anika Klaus, Silke Schwiebert, Peter Ruf, Horst Lindhofer, Holger N. Lode, Lena Andersch, Johannes H. Schulte, Angelika Eggert, Patrick Hundsdoerfer, Annette Künkele, Felix Zirngibl

**Affiliations:** ^1^ Department of Pediatric Oncology and Hematology, Berlin Institute of Health, Charité – Universitätsmedizin Berlin, Corporate member of Freie Universität Berlin, Humboldt – Universität zu Berlin and Berlin Institute of Health, Berlin, Germany; ^2^ Berlin Institute of Health (BIH), Berlin, Germany; ^3^ German Cancer Consortium (DKTK), Berlin, Germany; ^4^ Trion Research, Martinsried, Germany; ^5^ Pediatric Hematology and Oncology, University Medicine Greifswald, Greifswald, Germany; ^6^ German Cancer Research Center (DKFZ), Heidelberg, Germany; ^7^ Department of Pediatrics, HELIOS Klinikum Berlin Buch, Berlin, Germany

**Keywords:** tumor-vaccination, antibodies, immunotherapy, checkpoint inhibition, childhood cancer

## Abstract

**Introduction:**

Despite advances in treating high-risk neuroblastoma, 50-60% of patients still suffer relapse, necessitating new treatment options. Bispecific trifunctional antibodies (trAbs) are a promising new class of immunotherapy. TrAbs are heterodimeric IgG-like molecules that bind CD3 and a tumor-associated antigen simultaneously, whereby inducing a TCR-independent anti-cancer T cell response. Moreover, via their functional Fc region they recruit and activate cells of the innate immune system like antigen-presenting cells potentially enhancing induction of adaptive tumor-specific immune responses.

**Methods:**

We used the SUREK trAb, which is bispecific for GD2 and murine Cd3. Tumor-blind trAb and the monoclonal ch14.18 antibody were used as controls. A co-culture model of murine dendritic cells (DCs), T cells and a neuroblastoma cell line was established to evaluate the cytotoxic effect and the T cell effector function in vitro. Expression of immune checkpoint molecules on tumor-infiltrating T cells and the induction of an anti-neuroblastoma immune response using a combination of whole cell vaccination and trAb therapy was investigated in a syngeneic immunocompetent neuroblastoma mouse model (NXS2 in A/J background). Finally, vaccinated mice were assessed for the presence of neuroblastoma-directed antibodies. We show that SUREK trAb-mediated effective killing of NXS2 cells in vitro was strictly dependent on the combined presence of DCs and T cells.

**Results:**

Using a syngeneic neuroblastoma mouse model, we showed that vaccination with irradiated tumor cells combined with SUREK trAb treatment significantly prolonged survival of tumor challenged mice and partially prevent tumor outgrowth compared to tumor vaccination alone. Treatment led to upregulation of programmed cell death protein 1 (Pd-1) on tumor infiltrating T cells and combination with anti-Pd-1 checkpoint inhibition enhanced the NXS2-directed humoral immune response.

**Conclusion:**

Here, we provide first preclinical evidence that a tumor vaccination combined with SUREK trAb therapy induces an endogenous anti-neuroblastoma immune response reducing tumor recurrence. Furthermore, a combination with anti-Pd-1 immune checkpoint blockade might even further improve this promising immunotherapeutic concept in order to prevent relapse in high-risk neuroblastoma patients.

## Introduction

Bispecific trifunctional antibodies (trAbs) are a promising innovation in immunotherapy. Their anti-tumor efficacy is mediated by the innate and adaptive immune system. TrAbs are heterodimeric IgG-like molecules consisting of two different heavy and light chains and are engineered to have two antigen-binding sites in one molecule, enabling them to bind to a tumor-associated antigen and to CD3 from the T-cell receptor (TCR) complex simultaneously. This redirects T cells against the tumor and elicits a cytotoxic anti-tumor response independent of their TCR-specificity (1). In contrast to bispecific T cell engagers, trAbs contain an intact Fc region that can bind and activate Fc gamma receptor (FcγR)^+^ cells, such as monocytes or dendritic cells (DCs). This allows maturation of DCs that have taken up tumor antigens, which is required to trigger functional T- and B cell responses against potential tumor-specific neoantigens in tumor-draining lymph nodes *via* costimulatory signals like CD40, CD80 or CD86 (2). The SUREK trAb consists of the heterodimeric mouse IgG2a and rat IgG2b isotypes and has high affinity to FcγR I, IIa and III inducing activation of accessory immune cells like DCs (3). However, trAbs show only weak binding to NK cells *via* the FcγR IIIa when compared to humanized monoclonal IgG1 antibodies like ch14.18 and therefore are less effective in inducing antibody-dependent cellular cytotoxicity (1, 2). 

Neuroblastoma is the most common extracranial solid tumor in childhood and causes 15% of all tumor-related deaths in children (4). Half of all children diagnosed with neuroblastoma present with a high-risk disease and have a 5-year survival rate of less than 50% with most relapses occurring within the first two years (4–6). The overall survival (OS) of children with recurrent tumors is extremely low, ranging from 2 to 15%, denoting the need for new treatment options (5). The state-of-the art treatment schedule for primary neuroblastomas includes an induction treatment with 6-8 cycles of multi-agent chemotherapy, followed by surgery and radiation therapy. This is complemented by a consolidation therapy with high-dose myeloablative chemotherapy followed by autologous stem cell transplantation (6). The addition of the human-mouse chimeric ch14.18 monoclonal antibody (dinutuximab beta) targeting GD2, a disialoganglioside which is abundantly expressed on tumor cells of neuroectodermal origin, combined with granulocyte-macrophage colony-stimulating factor and interleukin 2 (IL2) improved two-year event-free survival (EFS) and OS of patients in the situation of minimal residual disease by 20% and 11%, respectively (7–9). Nevertheless, the 5-year EFS advantage decreased to 10% due to late relapses, as recently published (10). Even though immunotherapy has improved outcomes, long-term results still fall short of expectations (4).

T cells have surface markers which mediate inhibitory effector function maintaining self-tolerance and modulating immunity to prevent autoimmunity (11). These inhibitory molecules are also called “immune checkpoints” and can be exploited by malignant cells to inhibit or evade antitumor immune responses. Immune checkpoint inhibition therapies, targeting cytotoxic T-lymphocyte-associated Protein 4 (CTLA-4) or programmed cell death protein 1 (PD-1) have ameliorated outcomes of several different cancer types including metastatic melanoma and others (12, 13). Pediatric malignancies, such as neuroblastoma have not been part of this success (14). However, efficacy of immune checkpoint inhibitors might be enhanced when combined with other therapies. Srinivasan et al. successfully introduced a combination of checkpoint inhibitors with a whole cell vaccination in a preclinical neuroblastoma model (15). In order to be targetable by the GD2-directed SUREK trAb (GD2/Cd3), we need a tumor cell with an intact surfaceome. Thus, we also chose a whole cell vaccination approach.

Our group already showed that the GD2-directed SUREK trAb outperforms dinutuximab beta against neuroblastoma *in vivo* (6). We now set out to investigate whether cellular vaccination combined with SUREK trAb could effectively induce an endogenous adaptive anti-tumor immune response in a syngeneic neuroblastoma mouse model. Our approach represents a relapse prevention, comparable to the use of ch14.18 in the consolidation treatment of neuroblastoma. Additionally, after identification of an adequate candidate for checkpoint inhibition, we evaluated the benefit of a combination therapy of a cellular vaccine and the SUREK trAb with or without checkpoint inhibition regarding survival benefit and humoral immune response *in vivo*.

## Materials and methods

### Neuroblastoma cell line

The parental murine cell line NXS2 (16) was kindly provided by Holger N. Lode (HL) (University Medicine Greifswald, Germany). Cells were cultured in Dulbecco´s modified Eagle´s medium (DMEM, GIBCO, Darmstadt, Germany) supplemented with 10% fetal calf serum (FCS), 100 U/ml penicillin and 100 µg/ml streptomycin at 37°C in 5% CO_2_. Cultures were routinely tested for mycoplasma using the PlasmoTest™ Kit (Invitrogen GmbH, Darmstadt, Germany).

### T cells

T cells were isolated from spleens of 8-20-week-old male A/J mice *via* Pan T cell Isolation Kit II (Miltenyi Biotec, Bergisch Gladbach, Germany, cat#130-095-130) according to the manufacturer’s instructions. Purity of isolated murine Cd3+ cells was verified by flow cytometry and exceeded 90% (**Supplementary Figure 1**).

### Generation and characterization of dendritic cells

In order to generate bone marrow-derived DCs, bone marrow cells were isolated from *femura* and *tibiae* of 6-20-week-old male A/J mice. Cells were resuspended in Roswell Park Memorial Institute (RPMI) 1640 medium (GIBCO) and centrifuged at 1,200 rpm for 5 min. After being treated for 3 minutes with erythrocyte lysis buffer (pH=7.4; 0.1% KHCO3, 0.83% NH4Cl, and 0.00372% EDTA Titriplex) at room temperature, cells were washed with phosphate-buffered saline (PBS). Cells were adjusted to 0.5 x 10^6^ cells/ml and 1.5 x 10^6^ cells per well in a 6 well plate and were cultured in RPMI medium with 20% FCS, 100 U/ml penicillin and 100 µg/ml streptomycin, 1 mM ß-mercaptoethanol, 10 ng/ml granulocyte-macrophage colony-stimulating factor and 10 mM HEPES in a 6 well plate at 37°C in 5% CO_2_ for 7 days. On day 2 and 6, medium was exchanged. On day 4, cells were split. After one week of maturation, non-adherent cells were harvested, frozen in FCS with 10% DMSO (Carl Roth, Karlsruhe, Germany), and cryopreserved in liquid nitrogen until used for experiments. DCs were flow cytometrically characterized before they were used for experiments using LIVE/DEAD™ (Invitrogen, Carlsbad, CA, USA) to gate out dead cells and antibodies against Cd11c (cat#117334, Biolegend), Cd40 (cat#124624, Biolegend), Cd80 (cat#104741, Biolegend), Cd86 (cat#105012, Biolegend) and murine MHC class II (cat#107608, Biolegend) (**Supplementary Figure 2**). All samples were measured with an LSRFortessa™ (BD, 4 laser) and analysis was performed using FlowJo software (BD).

### Bispecific trifunctional antibodies

SUREK and TRBs012 antibodies (alphavirus E1/Cd3) were kindly provided by Lindis Biotech or Trion Research (Martinsried, Germany), where they were generated by quadroma technology and purified by affinity and ion exchange chromatography as previously described in detail (17).

### *In vitro* assessment of tumor cytotoxicity, cytokine release and T cell activation

For *in vitro* analysis, we used either parental NXS2 cells or NXS2 cells lentivirally transduced to express GFP and firefly luciferase (NXS2-GFP_ffluc) and sorted for GFP and GD2 expression (>80% positive cells; **Supplementary Figure 3**). NXS2 cells were co-cultured with T cells and dendritic cells at the ratio of either 1:10:1 or 1:10:3.3 depending on the experiment as indicated. To determine cytotoxicity, 30,000 NXS2-GFP_ffluc cells were seeded per well. After 1 h, freshly thawed DCs were added, again 2 h later, T cells were added. Subsequently, the co-culture was treated with trAb or control antibodies. Co-cultures were cultivated in RPMI media with 10% FCS + 100 U/ml penicillin and 100 µg/ml streptomycin + 1% non-essential-amino acids (Merck, Darmstadt, Germany) + 1 mM sodium pyruvat (Carl Roth) + 50mM ß-mercaptoethanol. TrAb-mediated cell death was quantified after 72 h in a biophotonic luciferase assay as previously described (18). Bioluminescence was measured after adding 28.6 µg of Xenolight D-Luciferin (PerkinElmer, Rodgau, Germany) per 150 µl of media after an exposure time of 180 s. Instrumental background was substracted before specific tumor cell lysis was calculated with the following formula: % specific lysis = [(RLU_tumor cells_) – (RLU_tumor cells + T cells + DCs +/- antibody_)]/(RLU_tumor cells_)*100% (6, 16). The release of interferon-y (Ifng) and Il2 was quantified in conditioned media of co-cultures using OptEIA enzyme-linked immunosorbent assays (BD, Heidelberg, Germany) after 24, 48 and 72 h according to the manufacturer’s instructions using murine Ifng (cat#555138) and murine Il2 (cat#555148). To this end, parental NXS2 cells were seeded in 96 well plates, and after a short rest (1 h) to allow target cells to settle, DCs were added, followed 2 h later by T cells (ratio of NXS2:T cells:DCs = 1:10:1) and trAbs or control antibodies. As previously described (18), T cell activation was flow cytometrically quantified 48 h after start of the co-culture (identical set up as for cytokine release assays) using LIVE/DEAD™ (Invitrogen) to gate out dead cells and antibodies against Cd3 (Biolegend, cat#100218), Cd4 (Biolegend, cat#1005599), Cd8a (Biolegend, cat#1000743), Cd69 (Biolegend, cat#104506), Pd-1 (Biolegend, cat#135210), lymphocyte activating gene 3 (Lag-3) (Biolegend, cat#125225) and Ctla-4 (Biolegend, cat#106306). All samples were measured with an LSRFortessa™ (BD) and analysis was performed using FlowJo software (BD). All assays were performed in technical as well as biological triplicates if not stated otherwise.

### Syngeneic mouse model

Male and female A/J mice were originally purchased from Jackson Laboratory (Bar Harbor, ME, USA) and bred in-house under pathogen-free conditions on a 12 h day/night cycle with free access to food and water, according to institutional guidelines approved *by Landesamt für Gesundheit und Soziales (LAGeSo, Berlin, Germany*, application numbers *G0386/17 and T0274/19)* and compliant with national and EU regulations for animal use in research.

### Analysis of tumor infiltrating T cells

Tumor infiltrating T cells were isolated as previously described (6). Inhibitory receptor expression was analyzed using LIVE/DEAD™ (Invitrogen) to gate out dead cells and antibodies against Cd3 (Biolegend, cat#100218), Cd4 (Biolegend, cat#1005599), Cd8a (Biolegend, cat#1000743), Pd-1 (Biolegend, cat#135210), lymphocyte activating gene 3 (Lag-3) (Biolegend, cat#125225) and Ctla-4 (Biolegend, cat#106306).

### Testing in syngeneic mouse model

For *in vivo* experiments, 10-22-week-old female animals were used. Mice were vaccinated with 1 x 10^5^ irradiated (100 Gray) NXS2 tumor cells intraperitoneally on day -21 and -7 immediately followed by intraperitoneal treatment with either 5 µg of SUREK trAb or respective control antibody (TRBs012 or ch14.18) diluted in 100 µl of PBS or 100 µl PBS alone. For combination with checkpoint inhibitor therapy, 250 µg of an anti-Pd1-antibody or PBS alone was given intraperitoneally on day -17, -13, -8 and -1. On day 0, animals were challenged with a subcutaneous injection of 1 x 10^6^ viable parental NXS2 cells. Tumor burden was recorded every other day using calipermetry of diameter in two dimensions. Tumor volume was calculated using the following formula: (length x width^2^)/2. When tumor burden reached a maximum size of 1,700 mm^3^, mice were euthanized. Blood was drawn from mice (*vena facialis*) 14 days after live tumor cell injection and shortly before animals had to be euthanized due to tumor burden. Blood samples were centrifuged at 4,000 rpm for 10 min at RT, serum collected and cryopreserved at -80°C until analysis.

### Analysis of humoral anti-tumor immune response

For quantification of tumor-directed antibodies in sera of mice, parental NXS2 cells were incubated with 100 µl serum harvested on day 14 or at final blood draw (dilution 1:30) for 1 h at 4°C. After two washing steps, a secondary fluorochrome-labelled antibody recognizing mouse IgG1 (Biolegend, cat#406610) or IgG2a (Biolegend, cat#407107) was added for another 30 min at 4°C. NXS2-specific binding of sera antibodies was quantified by flow cytometry with a LSRFortessa™ (BD) FlowJo software (BD) relative to NXS2 cells incubated with only the secondary antibody.

### Statistical analysis

All figures are representative of 3 experiments unless stated otherwise. Differences between testing groups in *in vitro* assays were analyzed using unpaired t test. Differences between testing groups in *in vivo* assays were compared using the Mann-Whitney test and the log-rank test. P values < 0.05 were considered statistically significant. Statistical analysis was performed using GraphPad Prism software V.9.3 (GraphPad Software, San Diego, CA, USA).

## Results

### SUREK trAb-mediated cytotoxicity against the murine neuroblastoma cell line NXS2 requires the presence of T and dendritic cells

**Figure 1:**
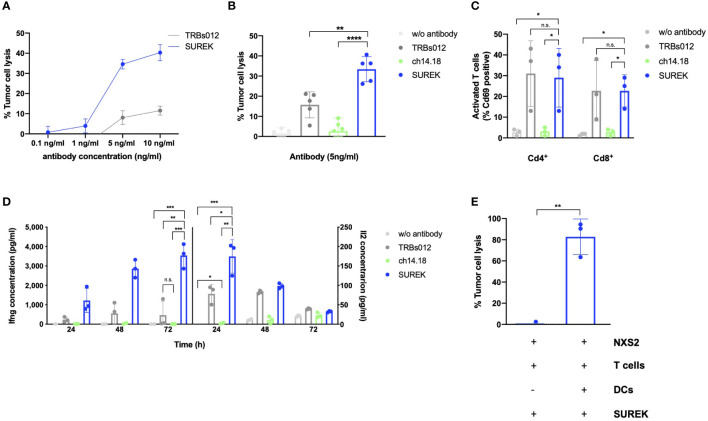
SUREK mediates a cytotoxic effect against the murine neuroblastoma cell line NXS2 dependent on T cells and dendritic cells. **(A)** The NXS2-GFP_ffluc cells were co-cultivated with T cells (TC) and dendritic cells (DCs) (ratio 1:10:1). After co-cultured cells had been settled for 3 hours, they were treated with the bispecific trifunctional antibodies (trAb) SUREK (GD2/Cd3) or TRBs012 (alphavirus E1/Cd3) in rising concentrations. Tumor cell lysis was determined by bioluminescent flux relative to an untreated co-culture after 72 hours (h). **(B)** NXS2-GFP_ffluc cells were co-cultivated as described in **(A)** and treated either with the SUREK trAb (GD2/Cd3), the monoclonal ch14.18 antibody (anti-GD2), the unspecific TRBs012 trAb (alphavirus E1/Cd3) or without any antibody. Tumor cell lysis was determined by bioluminescent flux relative to untreated NXS2 cells after 72 h Results are pooled medians of technical triplicates of five independent experiments. **(C)** Quantification of Cd69 expression on living Cd4^+^ and Cd8^+^ T cells after 48 h of co-culture as described in **(A)**. **(D)** After 24, 48 and 72 h of co-culture, concentration of the cytokines interferon gamma (Ifng) and interleukin-2 (Il2) in the conditioned media was determined by ELISA. One representative experiment with technical triplicates is shown. **(E)** NXS2-GFP_ffluc cells were co-cultivated with T cells together or without DCs (ratio 1:10:3.3). After cells had settled for 3 h, cells were treated with 10 ng/ml of SUREK and tumor cell lysis was determined after 72 h. Results are pooled medians of technical triplicates of three independent experiments. Student´s t test: *p<0.05; **p<0.01; ***p<0.001; ****p<0.0001; n.s., not significant.

In order to determine the minimal therapeutic dose of SUREK *in vitro*, we co-cultured the GD2^+^ murine neuroblastoma NXS2-GFP_ffluc cell line (**Supplementary Figure 3A**), primary murine T cells and DCs at a ratio of 1:10:1 and treated them with increasing amounts of SUREK and TRBs012 trAb ranging from 0.1 ng/ml to 10 ng/ml (**Figure 1A**). We detected a significant cytotoxicity (34.6% ± 2.9 lysed tumor cells) at SUREK trAb concentrations of 5 ng/ml, which plateaued at higher concentrations (5 ng/ml vs. 10 ng/ml, 34.6% ± 2.3 vs. 40.3% ± 4.0 lysed tumor cells). This was also seen for TRBs012 but to a lesser extend (5 ng/ml vs. 10 ng/ml, 8.07% vs. 11.52% lysed tumor cells).

Consequently, 5 ng/ml was used in all subsequent experiments. We used the TRBs012 trAb as well as the monoclonal ch14.18 antibody as controls at the same concentration as SUREK. They are either directed against Cd3 and a cancer-unrelated antigen (alphavirus E1; TRBs012) or monospecific against GD2 and lack a Cd3 binding moiety (ch14.18). To assess SUREK trAb’s efficacy to induce cytotoxicity, we co-cultured NXS2-GFP_ffluc cells, T cells and DCs at a ratio of 1:10:1. At an antibody concentration of 5 ng/ml, treatment with SUREK led to significantly higher tumor cell lysis in comparison to the unspecific control antibody TRBs012 (37.7% ± 2.54 vs. 14.6% ± 8.0 lysed tumor cells, p=0.009), while ch14.18 induced hardly any cytotoxic effect in this experimental setup (4.1% ± 1.6 lysed tumor cells; **Figure 1B**). This finding is reasonable as the ch14.18 mode of action is thought to be antibody-dependent cellular cytotoxicity (19).

Consistently, SUREK led to significantly higher upregulation of the activation marker Cd69 on Cd4^+^ (29.0% ± 14.2 positive cells, p=0.03) as well as on Cd8^+^ (21.5% ± 9.7 positive cells, p=0.03) T cells compared to treatment with ch14.18 antibody (Cd4^+^Cd69^+^: 3.0% ± 2.0 and Cd8^+^Cd69^+^: 2.6% ± 1.5; **Figure 1C**). The tumor-blind control trAb TRBs012 similarly activated T cells, independently of binding to a tumor antigen. Furthermore, T cells released higher amounts of Il2 and Ifng in co-cultures treated with SUREK trAb than with TRBs012 (174.6 pg/ml ± 35.2 vs. 78.2 pg/ml ± 19.8 and 3540.0 pg/ml ± 521.3 vs. 464.7 pg/ml ± 591.1), while ch14.18 induced almost no cytokine release (2.7 pg/ml ± 2.4 and 5.9 pg/ml ± 2.0) (**Figure 1D**).

Activated DCs provide a vital costimulatory signal for T cells to carry out their cytotoxic effect against tumor cells (1, 20). DCs themselves get activated *via* the interaction of their FcγR and the Fc fragment of the IgG2a and IgG2b isotypes of the SUREK trAb (1). Therefore, we co-cultured NXS2-GFP_ffluc cells, T cells with or without DCs in the presence of the SUREK trAb, in order to investigate the contribution of the Fc region of SUREK to tumor cell killing. To be able to point out the influence of DCs on tumor cell killing, we increased their amount in the co-culture from a tumor cell:T cell:DC ratio of 1:10:1 to 1:10:3.3. While almost 100% of tumor cells were killed in the presence of DCs, tumor cell lysis was completely abrogated when DCs were absent (**Figure 1E**). These data demonstrate that SUREK trAb treatment induces strong T cell activation and DC-dependent tumor cell killing consistent with results shown by Eissler et al. (21).

### Tumor cell vaccination in combination with SUREK treatment enhances overall and tumor-free survival of mice with aggressive syngeneic neuroblastoma transplants

**Figure 2:**
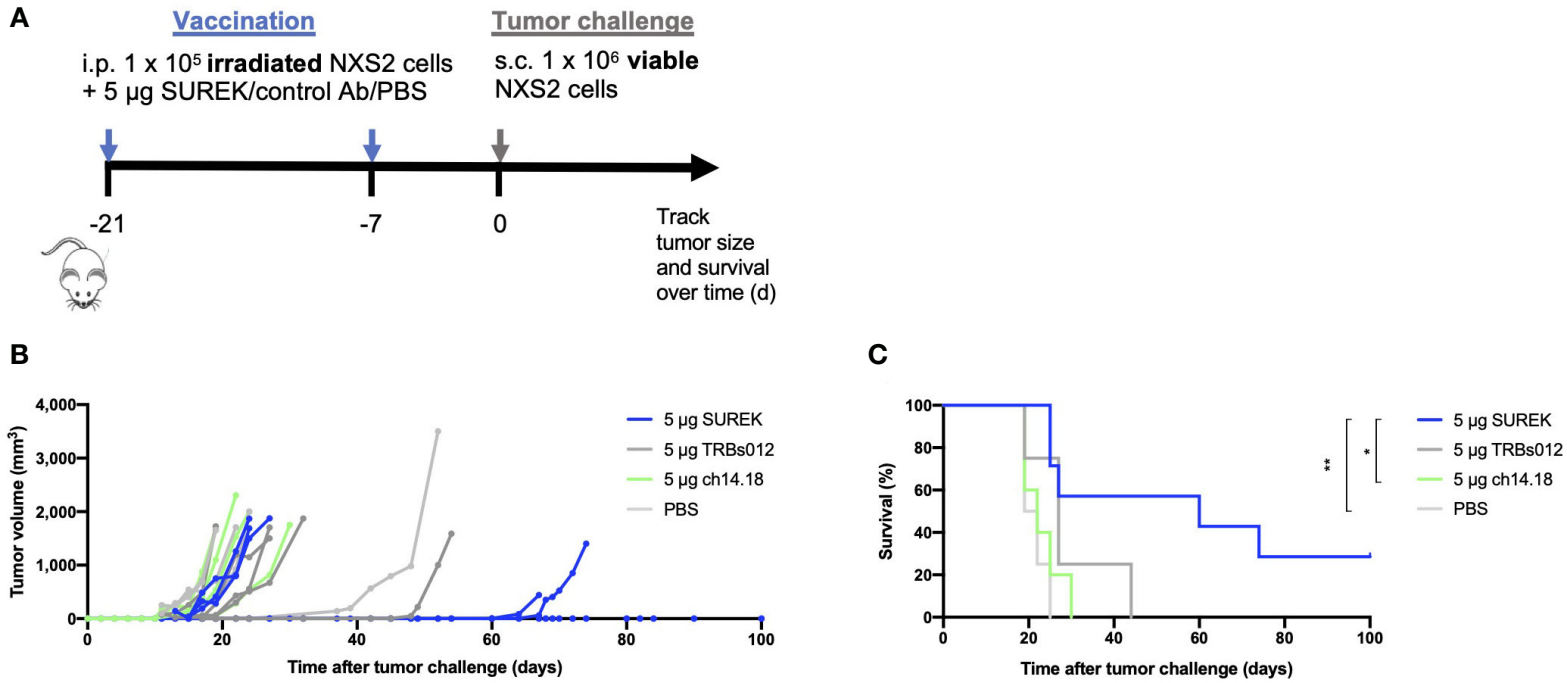
Tumor cell vaccination in combination with SUREK treatment enhances overall and tumor-free survival with aggressive syngeneic neuroblastoma transplants **(A)** Schematic representation of treatment and analysis. At day 21 and 7 before tumor challenge, A/J mice were vaccinated with intraperitoneal injections (i.p.) of 1 x 10^5^ irradiated NXS2 cells followed by i.p. injection of 5 µg of SUREK (n=7) or the control antibodies ch14.18 (n=5), TRBs012 (n=5) or PBS (n=4). On day 0, all mice were challenged with a subcutaneous injection of 1 x 10^6^ viable NXS2 cells. **(B)** Tumor size was checked every other day via calipermetry. **(C)** Survival of mice is shown in a Kaplan-Meier-Curve. Statistical analysis was done using log-rank test to compare different groups: *p<0.05; **p<0.01.

Having demonstrated *in vitro* that DCs are involved in the anti-tumor function of SUREK, we next sought to investigate whether anti-tumor immune responses by tumor vaccination could be enhanced by concomitant treatment with SUREK trAb in a syngeneic neuroblastoma mouse model. Following an established treatment schedule (22), we simultaneously injected irradiated NXS2 cells together with either SUREK, ch14.18, the tumor-blind TRBs012 trAb or PBS intraperitoneally 21 and 7 days before mice were challenged with a subcutaneous injection of live NXS2 cells (**Figure 2A**). Deppisch et al. already showed that 5 days after intravenous or subcutaneous administration, SUREK was not detectable anymore (23). None of the mice treated with tumor vaccination plus ch14.18, TRBs012 or PBS, rejected the tumor (**Figure 2B**). In contrast, subcutaneous challenge with living NXS2 tumor cells was completely rejected in two out of 7 mice (28.5%) during the surveillance period of 100 days treated concomitantly with tumor vaccination plus SUREK trAb. In two other mice the tumor growth was significantly delayed, resulting in a significantly enhanced survival in this treatment group compared to treatment groups having received ch14.18 or PBS (p=0.0144 and p=0.0032) (**Figure 2C**). In conclusion, while tumor cell vaccination alone was insufficient to prevent growth of subsequently injected tumor cells, combined treatment with SUREK prolonged tumor-free survival or even prevented tumor engraftment during the surveillance period of 100 days in our syngeneic mouse model.

### Combination therapy of SUREK and anti-Pd-1 leads to similar overall survival of mice, yet enhances humoral immunity in comparison to treatment with SUREK alone

**Figure 3:**
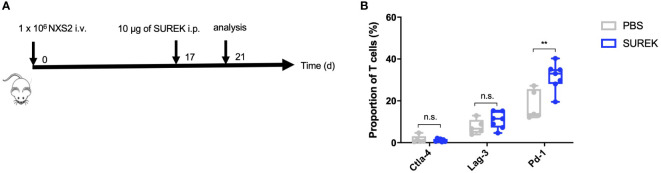
SUREK treatment leads to upregulation of Pd-1 on T cells. **(A)** A/J mice were injected with 1 x 106 NXS2 cells into a lateral tail vein (i.v.) and received one intraperitoneal (i.p.) injection with 10 µg of SUREK (GD2/Cd3) (n=7) or PBS (n=5) 17 days later. Four days after tumor injection, mice were sacrificed for analysis of tumor infiltrating T cells (TILs). **(B)** Expression of indicated checkpoint molecules on TILs of mice treated as described in **(A)**. Cd3 was used as a marker for T cells. Results are pooled from two independent experiments. Student´s t test: **p<0.01; n.s., not significant.

**Figure 4:**
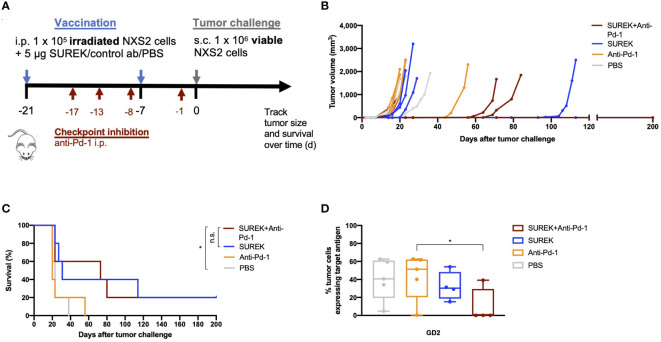
Combination therapy of SUREK and anti-Pd-1 does not significantly enhance overall survival of mice but shows a trend to immunogenicity in comparison to treatment with SUREK alone. **(A)** Schematic representation of treatment and analysis. A/J mice were vaccinated with intraperitoneal injection (i.p.) of 1 x 10^5^ irradiated NXS2 cells and a simultaneous i.p. injection of 5 µg of SUREK (n=5) or only PBS (n=5) on day -21 and -7. For combination therapy animals received anti-Pd-1 blocking antibody (n=5) on days -17, -13, -8, -1, control mice received PBS (n=5) or anti-Pd-1 blocking antibody (n=5) alone. On day 0 all mice were challenged with a subcutaneous injection of 1 x 10^6^ viable NXS2 cells which were injected subcutaneously into their right flank. **(B)** Tumor size was checked every other day via calipermetry. **(C)** Survival of mice is shown in a Kaplan-Meier-Curve. Statistical analysis was done using log-rank test to compare different groups: *p<0.05; n.s.: not significant. **(D)** Tumors that grew out after combination therapy were isolated and analyzed for GD2 surface expression using flow cytometry. Statistical analysis was done using Mann-Whitney test to compare different groups: *p<0.05.

As shown previously, splenocytes of mice vaccinated with B78-D14 tumor cells and treated with SUREK trAb upregulate the immune checkpoint Ctla-4, thereby suppressing anti-tumor T cell responses (22). Combining trAb therapy with checkpoint inhibition might therefore, further enhance anti-tumor T cell responses. To identify the most promising target for checkpoint inhibition, we analyzed upregulation of checkpoint molecules on tumor infiltrating T cells (TILs) after treatment with SUREK *in vivo*. After intravenous NXS2 cell injection, immunocompetent A/J mice predominantly developed liver metastases (19). Neuroblastoma originates from sympathoadrenergic progenitors and metastasis in neuroblastoma can be hematogenous or lymphogenic. Sites of hematogenous metastases are bone, bone marrow and liver. Especially relapses are often multilocular and spread hematogenously (24). Because we aim to treat relapsed or refractory high-risk neuroblastoma patients, we chose an intravenous tumor cell injection over a subcutaneous model to analyse TILs. Hence, animals were treated 17 days after tumor cell injection with a single dose of SUREK and T cells from spleen and liver metastases were harvested 4 days later to analyze checkpoint inhibitor expression *via* flow cytometry (**Figure 3A**). The gating strategy of TIL analysis is shown in **Supplementary Figure 4**. Surface expression of Pd-1 was significantly higher on TILs of mice treated with SUREK relative to the PBS-treated group (31.5% ± 6.6 vs. 18.0% ± 7.1 positive cells). As Pd-1 was upregulated the highest on murine TILs in comparison to Ctla-4 or Lag-3 (**Figure 3B**), we chose to block the Pd-1/Pd-l1 axis in order to enhance trAb-mediated anti-tumor immune response, as NXS2 cells are known to constitutively express Pd-l1 (25). We extended the treatment schedule described in **Figure 2A** by administering four doses of anti-Pd1 blocking antibody following an established treatment schedule for combination of the SUREK trAb and a checkpoint-inhibitor which was introduced by Deppisch et al. in a murine melanoma model (22) (**Figure 4A**). Again, we demonstrated improved survival of mice treated with concomitant tumor vaccination and SUREK compared to controls that received vaccination alone or combined with only checkpoint inhibition (**Figures 4B, C**). Yet, therapeutic efficacy was not improved by addition of anti-Pd-1 to SUREK therapy (1/5 vs. 1/5 surviving mice; **Figures 4B, C**). Since engraftment of subcutaneous tumors despite tumor vaccination and trAb +/- Pd-1 blockade might be due to the loss of GD2 expression, we analyzed cell surface GD2 expression on tumors isolated from treated mice using flow cytometry. While tumors from mice receiving combined treatment of tumor vaccination and SUREK tended (even though it was not significant) to express less GD2 compared to mice not receiving SUREK, GD2 expression was completely absent in 3 of 4 mice (75%) also receiving PD-1-blocking therapy (**Figure 4D**). Tumors growing out in mice that received anti-Pd-1 antibody also tend to express less Pd-l1 (**Supplementary Figure 5**). Interestingly, flow cytometric analysis showed a trend towards lower T cell infiltration in both groups receiving combined treatment with tumor vaccination and SUREK trAb with or without administration of Pd-1 blocking antibody (**Supplementary Figures 6A, B**). Overall, vaccination treatment with irradiated NXS2 and SUREK in combination with Pd-1 immune checkpoint blockade did not prolong mouse survival most likely due to GD2 antigen-loss.

### Combination therapy of SUREK with anti-Pd-1 antibody induces tumor-specific antibodies and Th2 memory response in mice

**Figure 5:**
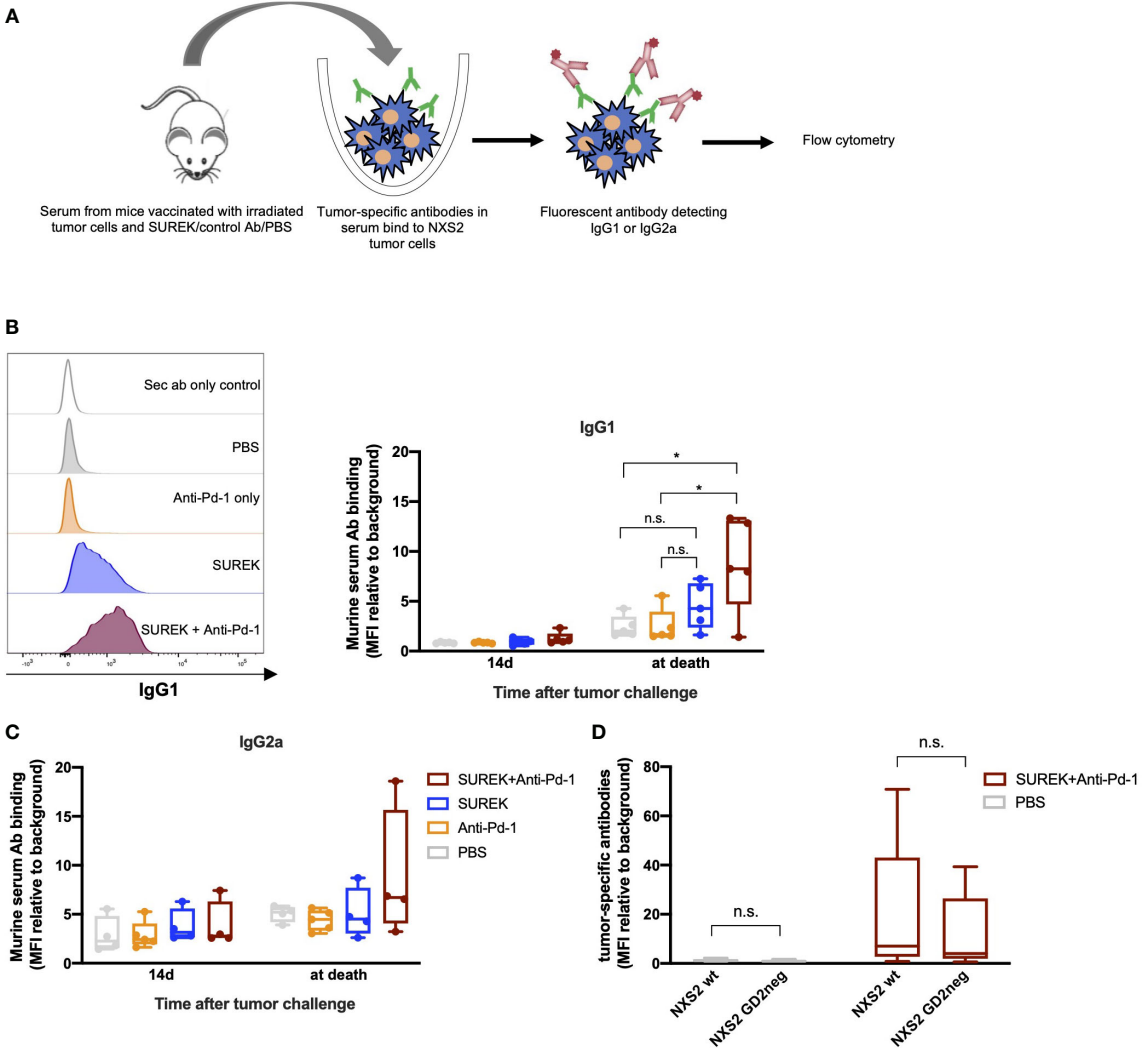
Combination therapy of SUREK with anti-Pd-1 antibody induces tumor-specific antibodies and T cell memory in mice. **(A)** Schematic representation of experiment and analysis. **(B)** Exemplary histograms (left panel) and quantification (mean fluorescence intensity (MFI)) relative to background, right panel) showing secondary IgG1 antibodies binding on NXS2 cells incubated with serum of mice that received the indicated treatment illustrated in Figure 4A. Statistical analysis was done using Student´s t test: *p<0.05; n.s., not significant. **(C)** Tumor-specific IgG2a antibodies in sera of vaccinated mice. Statistical analysis was done using Student´s t test. **(D)** Amount of NXS2 binding IgG1 antibodies in sera of mice. Experiment was conducted with GD2 positive and negative NXS2 cells (**Supplementary Figure 3A**).

We next investigated whether improved survival of mice receiving tumor vaccination in combination with SUREK coincided with the induction of an endogenous adaptive immune response against potential immunogenic epitopes expressed by the tumor cells. Therefore, we first assessed the presence of tumor-specific antibodies in sera of mice described in **Figures 4B, C** (**Figure 5A**). Whereas only a small amount of NXS2 tumor cell-specific IgG1 and IgG2a antibodies were detected in tumor-vaccinated mice receiving PBS or Pd-1 inhibitory antibody alone, SUREK-only treated mice developed a higher amount of IgG1 levels (**Figures 5B, C**). However, only vaccinated mice treated with the combination of SUREK and Pd-1 blockade, showed a significantly elevated IgG1 production compared to control groups (**Figure 5B**). The fact that binding of IgG1 antibodies in sera of mice to GD2 positive and negative NXS2 cells did not significantly differ, indicates a polyclonal humoral immune response against antigens other than GD2 (**Figure 5D**). In conclusion, we demonstrated that combination treatment of tumor vaccination with trAb elicited a humoral anti-tumor immune response that was enhanced under Pd-1 blockade.

## Discussion

Even though GD2-directed immunotherapy improved outcomes for high-risk or relapsed neuroblastoma patients, long-term follow-up dampened initial euphoria (9, 10, 26). We recently showed that GD2-directed bispecific trAbs are a promising new antibody therapy and that the SUREK trAb outperforms the monoclonal state-of-the-art ch14.18 antibody *in vitro* and *in vivo* (6). We now set out to investigate whether SUREK promotes the induction of a tumor-directed adaptive immune response against neuroblastoma *via* its functional Fc domain and how it compares to ch.14.18. In this study, we provide first evidence that SUREK induces a tumor-specific immunological memory, which was completely absent after tumor vaccination alone. The production of tumor-directed antibodies was increased by a combination therapy with anti-Pd-1 checkpoint inhibition. We show that the efficacy of SUREK is highly dependent on the presence of FcγR^+^ antigen-presenting cells (APCs) *in vitro* supposing that SUREK-mediated cytotoxicity depends on the presence of tumor cells, T cells and APC in our syngeneic murine neuroblastoma model as well.

Bispecific antibodies directed against GD2 and CD3 have already proven to be effective in murine neuroblastoma and melanoma xenograft models by Xu et al. (27). They could show release of Th1 cytokines, a GD2^+^-specific response *in vitro* and a prolonged survival from local relapse or metastasizing disease (27). A first phase I/II trial with the IgG-like GD2-directed hu3F8 bispecific antibody, which is an IgG1 containing a silenced Fc region and an anti-CD3 huOKT3-scFV domain linked to the carboxyl end of its light chain, is ongoing in neuroblastoma and other GD2^+^ tumor entities (26). Its silenced Fc region precludes the option to recruit and activate FcγR^+^ APCs (26–28). The lack of a Fc region prevents the induction of a long-term immunological memory that would allow tumor control beyond the presence of the bispecific antibody (20, 29, 30). With their functioning Fc fragment, TrAbs bring FcγR^+^ cells in close proximity to tumor and T cells, whereby activating T cells and inducing maturation of DCs. IL6 and IL12 release of DCs following treatment with the bispecific antibody BiUII (EpCAM/CD3) indicate activation of FcγR+ cells *via* the Fc fragment of the antibody (1). Treatment with the SUREK trAb could also induce high levels of Il12 in a syngeneic murine model for melanoma. T cells were only activated when DCs were present (shown by expression levels of Cd69 and Cd62L) and killing was absent when either SUREK or DCs were lacking (21). Interestingly, Thakur et al. could also show induction of tumor antigen-specific immune response *in vivo* and *in vitro* using activated T cells armed with anti-CD3 x anti-Her2/neu bispecific antibody lacking the Fc fragment. Multiple bispecific antibody armed T cell infusions may have resulted in immunogenic epitope spreading and the development of durable humoral and cellular memory response (31). Maturation of DCs is vital because it enables co-stimulation of T cells *via* CD40/CD40L or CD80-CD86/CD28. This interaction is critical to extend anti-tumor T cell responses beyond the specificity of the trAb by inducing functional T cell responses in the tumor draining lymph node (1, 20). We have demonstrated that a tumor cell vaccination alone was not sufficient to prevent engraftment and growth of a subcutaneous tumor cell mass in our syngeneic immunocompetent mouse model. In contrast, combining a tumor cell vaccination with SUREK trAb treatment prolonged survival of mice and even prevented tumor outgrowth during the surveillance period along with the presence of tumor-specific IgG antibodies in sera of treated mice. This was not observed when tumor vaccination was combined with ch14.18 or the tumor-blind trAb. The general therapy failure rate of 40 – 50% of vaccinated and SUREK treated animals might be due to a delayed immune response leading to tumor outgrowth in these animals. However, these results can still provide first indications that SUREK was not only mediating effector functions by cross-linking and activating T cells *via* its Cd3 and GD2 binding domains but also inducing adaptive immune responses against additional tumor antigens broadening up the immune response and having the potential to provide long-term tumor control by the induction of immunological memory.

Even though high-risk neuroblastoma is known to be little immunogenic and considered an immunologically cold tumor due to low mutational load and lack of MHC-I expression (32), it was also shown that relapsed neuroblastomas display a higher mutational burden as new somatic mutations are introduced by radiation and chemotherapy (32). Anti-neuroblastoma vaccination approaches have recently produced promising results. A GD2/GD3 vaccine plus oral administration of β-glucan as an additional adjuvant elicited robust anti-GD2 and -GD3 antibody responses in patients with high-risk neuroblastoma (33). High anti-GD2-IgG1 titer was associated with improved survival (33). In our study, tumor-specific IgG1 antibodies in mice having received combined treatment of tumor vaccination and SUREK, but not vaccination alone or combined treatment with the monoclonal antibody ch14.18, was associated with survival benefit. These trAb-induced tumor-specific antibodies are directed against a various set of tumor antigens. Ruf et al. showed a similar induction of a humoral immune response by SUREK in a preclinical melanoma model (34). So far there is no available literature about potentially immunogenic NXS2-specific tumor associated antigens. Although, level of tumor-specific antibodies in mice receiving additional Pd-1 blocking antibodies was significantly increased, this did not translate into prolonged survival. The fact that the majority of progressing tumors has lost GD2 expression in this treatment group indicates that immune pressure was higher, which eventually resulted in the outgrowth of antigen-loss variants. Similar mechanisms have been shown for sarcoma mouse models (35), small cell lung cancer (36) or glioblastoma (37). Although the presence of tumor-specific IgG antibodies indicates the induction of an adaptive immune response against additional tumor antigens, tumor cell destruction in some of the mice might have been prevented by immunosuppressive mechanisms other than installed by the Pd-1/Pd-l1 axis. A recent study by Webb et al. demonstrated that NXS2-tumor-bearing A/J mice had a significantly higher splenic Treg count than tumor-free mice. Furthermore, CD4^+^ T cells in the tumor were composed mainly of Treg cells, indicating an important role of immune suppression by this cell type (38). Although expression of Ctla-4 was hardly induced by trAbs on total Cd3^+^ cells in the tumor, there is a chance to further improve efficacy of our combination therapy of tumor vaccination and SUREK trAb by inhibiting Tregs by CTLA-4 immune checkpoint blockade as the combination with the checkpoint inhibitor anti-CTLA-4 proved to be superior to anti-PD-1 monotherapy in a preclinical neuroblastoma model (39). While *in vivo* significance of immune checkpoint molecules in neuroblastoma remains uncertain, there are promising results *in vitro* (14, 40). Interaction of immune checkpoint molecules like CTLA-4, LAG-3 or PD-1 with their ligands B7.1/B7.2 on antigen-presenting cells, fibrinogen-like protein-1 and Galectin-3 or PD-L1 on tumor cells, respectively, enhance immune escape of tumor cells (41). Consequently, checkpoint-inhibition is beneficial to boost tumor-reactive T cell function in several different tumor entities (22, 42). The combination of cyclophosphamide and anti-Pd-1 was shown to be effective in a pre-clinical neuroblastoma mouse model (43). In Neuroblastoma, expression of PD-L1 is both constitutive and inducible by different mechanisms such as IFNG-induced signaling, with expression levels ranging from 14% to 70% in patients (14, 44, 45). Consequently, the combination of a Myc-inhibited- or Id2 knockdown-whole tumor cell vaccination and the checkpoint inhibitors anti-PD-1 and anti-CTLA-4 was successfully tested in a preclinical anti-neuroblastoma vaccination attempt when immunogenicity of tumors was enhanced and a long-term immune memory induced (15, 46). Other preclinical publications showed their efficacy against neuroblastoma in combination with dinutuximab beta (47) or with radiation and an anti-CD40 antibody (40). The efficacy of our treatment schedule might be enhanced by adding more doses of checkpoint-inhibitor and/or additional vaccinations. Also, applying the subcutaneous tumor challenge 7 days after the last vaccination might be too short for the induction of a robust immune response.

One advantage of our study is that we showed efficacy of our treatment in an immunocompetent syngeneic neuroblastoma mouse model. In this model, assessment of immunotherapies is most beneficial as there is no species-mismatch of tumor, stroma and immune cells like in models with human tumor xenografts, providing the basis to analyze the full effector function potential by SUREK trAb that requires the tripartite interplay of tumor, T cells and DCs. However, the A/J strain might not be optimal due to its Th2-bias and relatively high immunogenicity (48). Prospectively, the SUREK-mediated vaccination effect might also be tested in a less immunogenic GD2-expressing neuroblastoma mouse model with a murine TH-MYCN transgenic neuroblastoma cell line (9464D-GD2) established by Voeller et al. (40). Whereas we predominantly see a tumor-directed IgG1 production, Ruf et al. and Deppisch et al. showed a shift towards a Th1 response characterized by the production of tumor-directed IgG2a in a C57BL/6 mouse model, which is known to show a predominant Th1 response (22, 34). Th1 cells produce IFNG and IL2 among other cytokines, thereby activating macrophages and inducing a cell-mediated immunity, whereas a Th2 response is known to induce a humoral immunity with B cell proliferation and antibody production (49). Furthermore, Th2 cells are known to secrete predominantly IL4, which suppresses the development of Th1 cells on the one hand and directly blocks the synthesis of IFNG in human mononuclear cells with its cytostatic, pro-apoptotic and immune-provoking effects on the other hand (49, 50). To date, it is not quite clear whether a Th2-dominant immune response is beneficial or detrimental for tumor growth (51). However, there is evidence, that IL4–secreting Th2 cells, inhibit proliferation of cytotoxic T cells and induce an M2 phenotype in tumor-associated macrophages, promoting tumor cell growth and invasion (52).

In summary, we provide first evidence that the GD2-directed SUREK trAb is not only potent to mediate TCR-independent anti-tumor T cell responses, but also induces an anti-tumor vaccination effect in a murine neuroblastoma model that prolonged survival of tumor-challenged mice. Anti-Pd-1 immune checkpoint blockade further enhanced immune pressure on tumor cells resulting in tumor progression due to target antigen loss. Combining tumor vaccination and SUREK trAb treatment with additional checkpoint inhibitors might further improve anti-tumor effector function and could translate into the clinic as a prophylactic therapy option for children with minimal residual disease.

## Data availability statement

The original contributions presented in the study are included in the article/**Supplementary Material**. Further inquiries can be directed to the corresponding author.

## Ethics statement

The animal study was reviewed and approved by Landesamt für Gesundheit und Soziales, Berlin, Germany.

## Author contributions

SI co-conceived the study, designed and performed experiments, interpreted data and wrote the manuscript. FZ and AKü jointly conceived the study, designed experiments, interpreted data, wrote and revised the manuscript. FZ and AKü contributed equally to this work. LG, LA, ML, AKl, SS, and KA designed and performed experiments and interpreted data. HLo provided NXS2 cells, PR and HLi provided bispecific trifunctional antibodies and interpreted data. AE, JS and PH co-conceived the study and interpreted data. All authors contributed to the article and approved the submitted version.

## References

[1] Zeidler R Reisbach G Wollenberg B Lang S Chaubal S Schmitt B. Simultaneous activation of T cells and accessory cells by a new class of intact bispecific antibody results in efficient tumor cell killing. J Immunol (1999) 163(3):1246–52. 10415020

[2] Zeidler R Mysliwietz J Csanady M Walz A Ziegler I Schmitt B. The fc-region of a new class of intact bispecific antibody mediates activation of accessory cells and NK cells and induces direct phagocytosis of tumour cells. Br J Cancer (2000) 83(2):261–6. doi: 10.1054/bjoc.2000.1237 PMC2363488 10901380

[3] Dekkers G Bentlage AEH Stegmann TC Howie HL Lissenberg-Thunnissen S Zimring J. Affinity of human IgG subclasses to mouse fc gamma receptors. mAbs (2017) 9(5):767–73. doi: 10.1080/19420862.2017.1323159 PMC5524164 28463043

[4] Zafar A Wang W Liu G Wang X Xian W McKeon F. Molecular targeting therapies for neuroblastoma: Progress and challenges. Med Res Rev (2021) 41(2):961–1021. doi: 10.1002/med.21750 33155698 PMC7906923

[5] Herd F Basta NO McNally RJQ Tweddle DA. A systematic review of re-induction chemotherapy for children with relapsed high-risk neuroblastoma. Eur J Cancer (2019) 111:50–8. doi: 10.1016/j.ejca.2018.12.032 PMC6458963 30822684

[6] Zirngibl F Ivasko SM Grunewald L Klaus A Schwiebert S Ruf P. GD2-directed bispecific trifunctional antibody outperforms dinutuximab beta in a murine model for aggressive metastasized neuroblastoma. J Immunother Cancer (2021) 9(7):e002923. doi: 10.1136/jitc-2021-002923 34285106 PMC8292814

[7] Ladenstein R Potschger U Valteau-Couanet D Luksch R Castel V Ash S. Investigation of the role of dinutuximab beta-based immunotherapy in the SIOPEN high-risk neuroblastoma 1 trial (HR-NBL1). Cancers (Basel) (2020) 12(2):309. doi: 10.3390/cancers12020309 32013055 PMC7072500

[8] Ladenstein R Potschger U Valteau-Couanet D Luksch R Castel V Yaniv I. Interleukin 2 with anti-GD2 antibody ch14.18/CHO (dinutuximab beta) in patients with high-risk neuroblastoma (HR-NBL1/SIOPEN): A multicentre, randomised, phase 3 trial. Lancet Oncol (2018) 19(12):1617–29. doi: 10.1016/S1470-2045(18)30578-3 30442501

[9] Yu AL Gilman AL Ozkaynak MF London WB Kreissman SG Chen HX. Anti-GD2 antibody with GM-CSF, interleukin-2, and isotretinoin for neuroblastoma. N Engl J Med (2010) 363(14):1324–34. doi: 10.1056/NEJMoa0911123 PMC3086629 20879881

[10] Yu AL Gilman AL Ozkaynak MF Naranjo A Diccianni MB Gan J. Long-term follow-up of a phase III study of ch14.18 (Dinutuximab) + cytokine immunotherapy in children with high-risk neuroblastoma: COG study ANBL0032. Clin Cancer Res (2021) 27(8):2179–89. doi: 10.1158/1078-0432.CCR-20-3909 PMC8046731 33504555

[11] Pardoll DM. The blockade of immune checkpoints in cancer immunotherapy. Nat Rev Cancer (2012) 12(4):252–64. doi: 10.1038/nrc3239 PMC4856023 22437870

[12] Wilson RAM Evans TRJ Fraser AR Nibbs RJB. Immune checkpoint inhibitors: New strategies to checkmate cancer. Clin Exp Immunol (2018) 191(2):133–48. doi: 10.1111/cei.13081 PMC5758374 29139554

[13] Wolchok JD Chiarion-Sileni V Gonzalez R Rutkowski P Grob JJ Cowey CL. Overall survival with combined nivolumab and ipilimumab in advanced melanoma. N Engl J Med (2017) 377(14):1345–56. doi: 10.1056/NEJMoa1709684 PMC5706778 28889792

[14] Wienke J Dierselhuis MP Tytgat GAM Kunkele A Nierkens S Molenaar JJ. The immune landscape of neuroblastoma: Challenges and opportunities for novel therapeutic strategies in pediatric oncology. Eur J Cancer (2021) 144:123–50. doi: 10.1016/j.ejca.2020.11.014 33341446

[15] Srinivasan P Wu X Basu M Rossi C Sandler AD. PD-L1 checkpoint inhibition and anti-CTLA-4 whole tumor cell vaccination counter adaptive immune resistance: A mouse neuroblastoma model that mimics human disease. PloS Med (2018) 15(1):e1002497. doi: 10.1371/journal.pmed.1002497 29377881 PMC5788338

[16] Greene LA Shain W Chalazonitis A Breakfield X Minna J Coon HG. Neuronal properties of hybrid neuroblastoma X sympathetic ganglion cells. Proc Natl Acad Sci U S A (1975) 72(12):4923–7. doi: 10.1073/pnas.72.12.4923 PMC388845 1745

[17] Ruf P Jager M Ellwart J Wosch S Kusterer E Lindhofer H. Two new trifunctional antibodies for the therapy of human malignant melanoma. Int J Cancer (2004) 108(5):725–32. doi: 10.1002/ijc.11630 14696099

[18] Ali S Toews K Schwiebert S Klaus A Winkler A Grunewald L. Tumor-derived extracellular vesicles impair CD171-specific CD4(+) CAR T cell efficacy. Front Immunol (2020) 11:531. doi: 10.3389/fimmu.2020.00531 32296437 PMC7137471

[19] Zeng Y Fest S Kunert R Katinger H Pistoia V Michon J. Anti-neuroblastoma effect of ch14.18 antibody produced in CHO cells is mediated by NK-cells in mice. Mol Immunol (2005) 42(11):1311–9 doi: 10.1016/j.molimm.2004.12.018. 15950727

[20] Hess J Ruf P Lindhofer H. Cancer therapy with trifunctional antibodies: linking innate and adaptive immunity. Future Oncol (2012) 8(1):73–85. doi: 10.2217/fon.11.138 22149036

[21] Eissler N Mysliwietz J Deppisch N Ruf P Lindhofer H Mocikat R. Potential of the trifunctional bispecific antibody surek depends on dendritic cells: Rationale for a new approach of tumor immunotherapy. Mol Med (2013) 19(1):54–61. doi: 10.2119/molmed.2012.00140 23552725 PMC3646096

[22] Deppisch N Ruf P Eissler N Lindhofer H Mocikat R. Potent CD4+ T cell-associated antitumor memory responses induced by trifunctional bispecific antibodies in combination with immune checkpoint inhibition. Oncotarget (2017) 8(3):4520–9. doi: 10.18632/oncotarget.13888 PMC5354850 27966460

[23] Deppisch N Ruf P Eissler N Neff F Buhmann R Lindhofer H. Efficacy and tolerability of a GD2-directed trifunctional bispecific antibody in a preclinical model: Subcutaneous administration is superior to intravenous delivery. Mol Cancer Ther (2015) 14(8):1877–83. doi: 10.1158/1535-7163.MCT-15-0156 26063765

[24] Matthay KK Maris JM Schleiermacher G Nakagawara A Mackall CL Diller L. Neuroblastoma. Nat Rev Dis Primers (2016) 2:16078. doi: 10.1038/nrdp.2016.78 27830764

[25] Rigo V Emionite L Daga A Astigiano S Corrias MV Quintarelli C. Combined immunotherapy with anti-PDL-1/PD-1 and anti-CD4 antibodies cures syngeneic disseminated neuroblastoma. Sci Rep (2017) 7(1):14049. doi: 10.1038/s41598-017-14417-6 29070883 PMC5656588

[26] Modak S. Study of the safety and efficacy of humanized 3F8 bispecific antibody (Hu3F8-BsAb) in patients with Relapsed/Refractory neuroblastoma, osteosarcoma and other solid tumor cancers 2019 . Available at: https://ClinicalTrials.gov/show/NCT03860207 (Accessed July 2022).

[27] Xu H Cheng M Guo H Chen Y Huse M Cheung NK. Retargeting T cells to GD2 pentasaccharide on human tumors using bispecific humanized antibody. Cancer Immunol Res (2015) 3(3):266–77. doi: 10.1158/2326-6066.CIR-14-0230-T PMC4351131 25542634

[28] Park JA Cheung NV. Targets and antibody formats for immunotherapy of neuroblastoma. J Clin Oncol (2020) 38(16):1836–48. doi: 10.1200/JCO.19.01410 PMC7255979 32167865

[29] Eissler N Ruf P Mysliwietz J Lindhofer H Mocikat R. Trifunctional bispecific antibodies induce tumor-specific T cells and elicit a vaccination effect. Cancer Res (2012) 72(16):3958–66. doi: 10.1158/0008-5472.CAN-12-0146 22745368

[30] Kontermann RE Brinkmann U. Bispecific antibodies. Drug Discov Today (2015) 20(7):838–47. doi: 10.1016/j.drudis.2015.02.008 25728220

[31] Thakur A Rathore R Kondadasula SV Uberti JP Ratanatharathorn V Lum LG. Immune T cells can transfer and boost anti-breast cancer immunity. Oncoimmunology (2018) 7(12):e1500672. doi: 10.1080/2162402X.2018.1500672 30524893 PMC6279339

[32] Schramm A Köster J Assenov Y Althoff K Peifer M Mahlow E. Mutational dynamics between primary and relapse neuroblastomas. Nat Genet (2015) 47(8):872–7. doi: 10.1038/ng.3349 26121086

[33] Cheung IY Cheung NV Modak S Mauguen A Feng Y Basu E. Survival impact of anti-GD2 antibody response in a phase II ganglioside vaccine trial among patients with high-risk neuroblastoma with prior disease progression. J Clin Oncol (2021) 39(3):215–26. doi: 10.1200/JCO.20.01892 PMC8253584 33326254

[34] Ruf P Schafer B Eissler N Mocikat R Hess J Ploscher M. Ganglioside GD2-specific trifunctional surrogate antibody surek demonstrates therapeutic activity in a mouse melanoma model. J Transl Med (2012) 10:219. doi: 10.1186/1479-5876-10-219 23134699 PMC3543252

[35] DuPage M Mazumdar C Schmidt LM Cheung AF Jacks T. Expression of tumour-specific antigens underlies cancer immunoediting. Nature (2012) 482(7385):405–9. doi: 10.1038/nature10803 PMC3288744 22318517

[36] Anagnostou V Smith KN Forde PM Niknafs N Bhattacharya R White J. Evolution of neoantigen landscape during immune checkpoint blockade in non-small cell lung cancer. Cancer Discov (2017) 7(3):264–76. doi: 10.1158/2159-8290.CD-16-0828 PMC5733805 28031159

[37] O’Rourke DM Nasrallah MP Desai A Melenhorst JJ Mansfield K Morrissette JJD. A single dose of peripherally infused EGFRvIII-directed CAR T cells mediates antigen loss and induces adaptive resistance in patients with recurrent glioblastoma. Sci Transl Med (2017) 9(399):eaaa0984. doi: 10.1126/scitranslmed.aaa0984 28724573 PMC5762203

[38] Webb ER Lanati S Wareham C Easton A Dunn SN Inzhelevskaya T. Immune characterization of pre-clinical murine models of neuroblastoma. Sci Rep (2020) 10(1):16695. doi: 10.1038/s41598-020-73695-9 33028899 PMC7541480

[39] Shirinbak S Chan RY Shahani S Muthugounder S Kennedy R Hung LT. Combined immune checkpoint blockade increases CD8+CD28+PD-1+ effector T cells and provides a therapeutic strategy for patients with neuroblastoma. Oncoimmunology (2021) 10(1):1838140. doi: 10.1080/2162402X.2020.1838140 33489468 PMC7801125

[40] Voeller J Erbe AK Slowinski J Rasmussen K Carlson PM Hoefges A. Combined innate and adaptive immunotherapy overcomes resistance of immunologically cold syngeneic murine neuroblastoma to checkpoint inhibition. J Immunother Cancer (2019) 7(1):344. doi: 10.1186/s40425-019-0823-6 31810498 PMC6898936

[41] Tu L Guan R Yang H Zhou Y Hong W Ma L. Assessment of the expression of the immune checkpoint molecules PD-1, CTLA4, TIM-3 and LAG-3 across different cancers in relation to treatment response, tumor-infiltrating immune cells and survival. Int J Cancer (2020) 147(2):423–39. doi: 10.1002/ijc.32785 31721169

[42] Rizvi NA Hellmann MD Snyder A Kvistborg P Makarov V Havel JJ. Cancer immunology. mutational landscape determines sensitivity to PD-1 blockade in non-small cell lung cancer. Science (2015) 348(6230):124–8. doi: 10.1126/science.aaa1348 PMC4993154 25765070

[43] Webb ER Moreno-Vicente J Easton A Lanati S Taylor M James S. Cyclophosphamide depletes tumor infiltrating T regulatory cells and combined with anti-PD-1 therapy improves survival in murine neuroblastoma. iScience (2022) 25(9):104995. doi: 10.1016/j.isci.2022.104995 36097618 PMC9463572

[44] Boes M Meyer-Wentrup F. TLR3 triggering regulates PD-L1 (CD274) expression in human neuroblastoma cells. Cancer Lett (2015) 361(1):49–56 doi: 10.1016/j.canlet.2015.02.027 25697485

[45] Saletta F Vilain RE Gupta AK Nagabushan S Yuksel A Catchpoole D. Programmed death-ligand 1 expression in a Large cohort of pediatric patients with solid tumor and association with clinicopathologic features in neuroblastoma. JCO Precis Oncol (2017) 1:1–12. doi: 10.1200/PO.16.00049 35172499

[46] Wu X Nelson M Basu M Srinivasan P Lazarski C Zhang P. MYC oncogene is associated with suppression of tumor immunity and targeting myc induces tumor cell immunogenicity for therapeutic whole cell vaccination. J Immunother Cancer (2021) 9(3):e001388. doi: 10.1136/jitc-2020-001388 33757986 PMC7993333

[47] Siebert N Zumpe M Juttner M Troschke-Meurer S Lode HN. PD-1 blockade augments anti-neuroblastoma immune response induced by anti-GD2 antibody ch14.18/CHO. Oncoimmunology (2017) 6(10):e1343775. doi: 10.1080/2162402X.2017.1343775 29123953 PMC5665083

[48] Mitaka K Miyazaki Y Yasui M Furuie M Miyake S Inase N. Th2-biased immune responses are important in a murine model of chronic hypersensitivity pneumonitis. Int Arch Allergy Immunol (2011) 154(3):264–74. doi: 10.1159/000321114 20861649

[49] Noble A Kemeny DM. Interleukin-4 enhances interferon-gamma synthesis but inhibits development of interferon-gamma-producing cells. Immunology (1995) 85(3):357–63. PMC1383907 7558122

[50] Kursunel MA Esendagli G. The untold story of IFN-γ in cancer biology. Cytokine Growth Factor Rev (2016) 31:73–81. doi: 10.1016/j.cytogfr.2016.07.005 27502919

[51] Ellyard JI Simson L Parish CR. Th2-mediated anti-tumour immunity: friend or foe? Tissue Antigens (2007) 70(1):1–11. doi: 10.1111/j.1399-0039.2007.00869.x 17559575

[52] DeNardo DG Barreto JB Andreu P Vasquez L Tawfik D Kolhatkar N. CD4(+) T cells regulate pulmonary metastasis of mammary carcinomas by enhancing protumor properties of macrophages. Cancer Cell (2009) 16(2):91–102. doi: 10.1016/j.ccr.2009.06.018 19647220 PMC2778576

