# Supplementary material for: Combination of GD2-directed bispecific trifunctional antibody therapy with Pd-1 immune checkpoint blockade induces anti-neuroblastoma immunity in a syngeneic mouse model
Source: Front Immunol. 2023 Jan 9;13:1023206. doi: 10.3389/fimmu.2022.1023206 (PMC9869131; doi:10.3389/fimmu.2022.1023206)
Supplement: Supplementary file 1 [file Image_1.pdf]

Online Supplementary Material

Ivasko SM *et al*, Combination of GD2-directed bispecific trifunctional antibody therapy with Pd-1 immune checkpoint blockade induces anti-neuroblastoma immunity in a syngeneic mouse model

Supplementary Figures

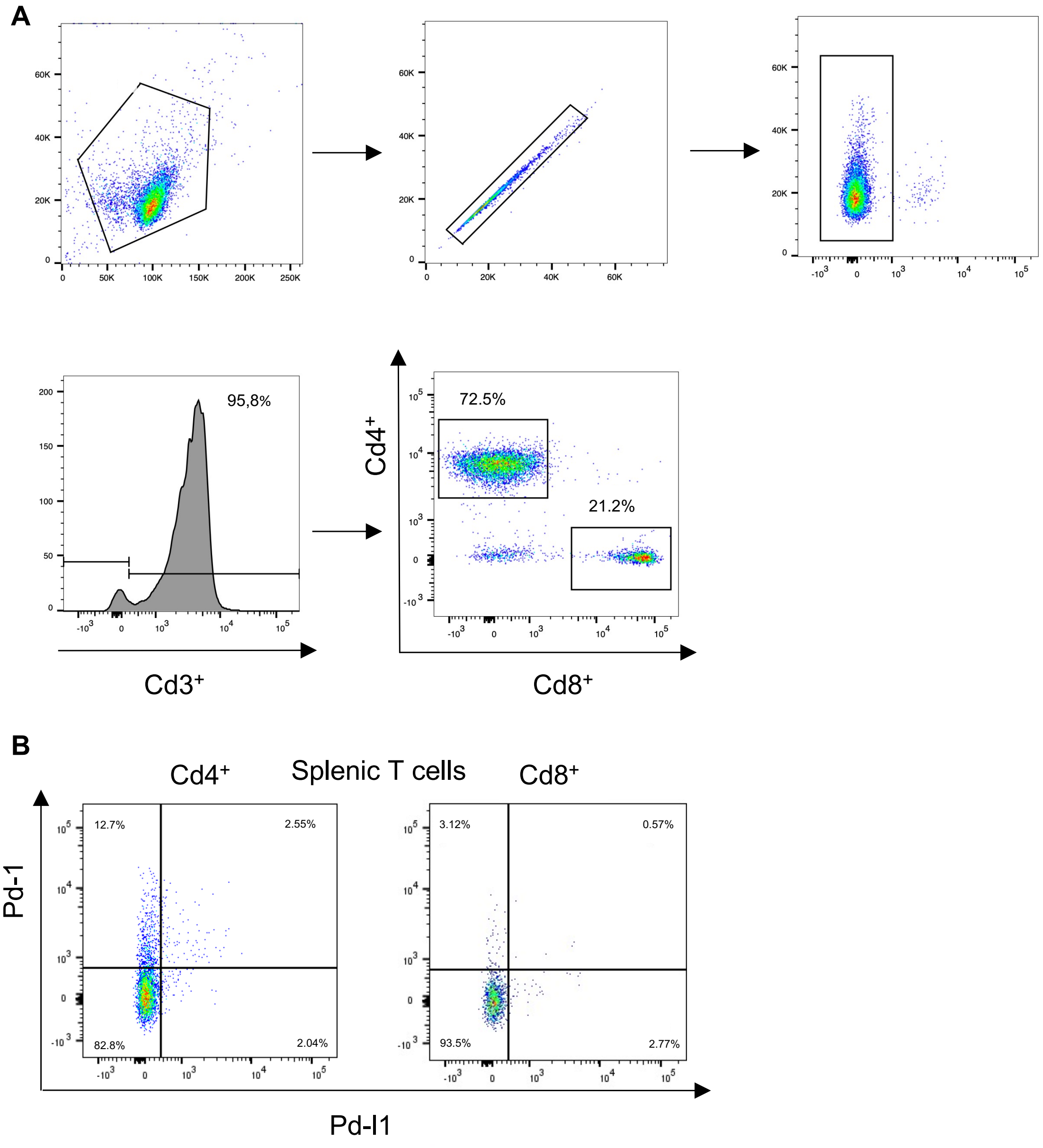

Supplementary Figure 1. Characterization of T cells prior to *in vitro* co-cultivation experiments. (A) Murine splenocytes were extracted from spleens of A/J mice and total T cells were isolated via negative selection. Purity of extraction is shown by $Cd3^+$, $Cd4^+$ and $Cd8^+$ staining. **(B)** Pd-1 expression of indicated T cell subpopulations isolated from an untreated A/J mice.

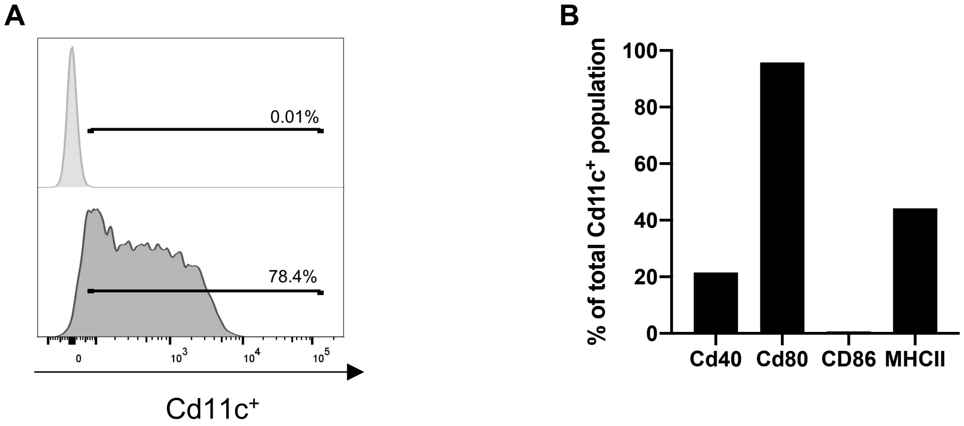

Supplementary Figure 2. Characterization of naïve dendritic cells isolated from a naïve A/J mouse and used for *in vitro* co-cultivation experiments. (**A**) Purity of isolated dendritic cells was analyzed by $CD11c^+$ staining. **(B)** Isolated dendritic cells were analyzed for their expression of maturation specific markers.

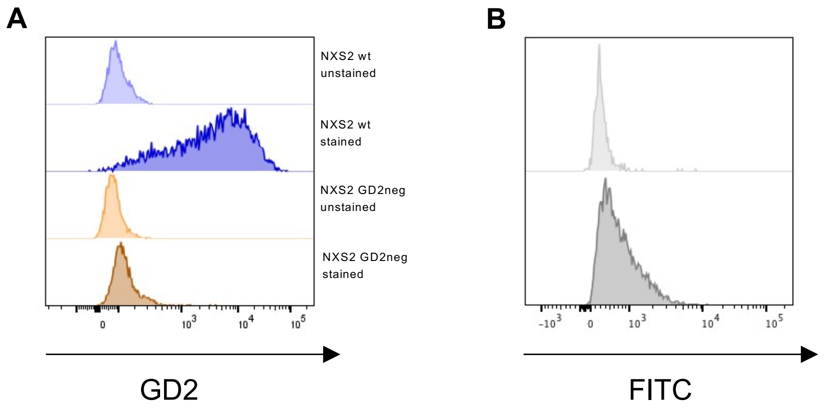

Supplementary Figure 3. Characterization of naïve murine NXS2 cells before treatment. (A) The original murine NXS2 cell line expresses high amounts of GD2. We generated a cell line which is negative for GD2 by injecting the wild type (wt) cell line into A/J mice which were then treated with SUREK. Consequently, tumor cells downregulated GD2 and were then taken in culture. **(B)** For Luciferase-based-killing-assays we stably transduced NXS2 wt cell line with a GFP_ffluc construct (NXS2-GFP_ffluc, dark grey), which can be seen by a brighter signal of FITC labelled cells in flow cytometry analysis in comparison to untransduced NXS2 cells.

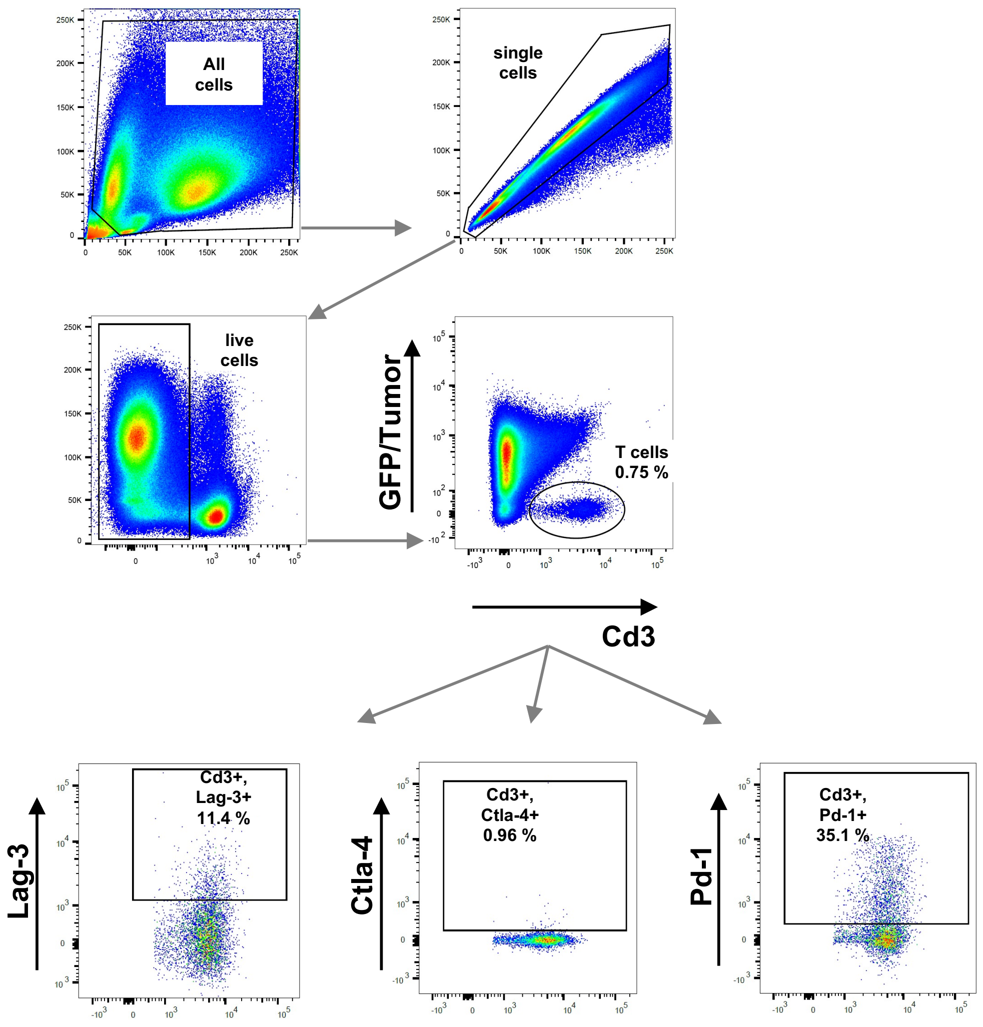

Supplementary Figure 4. Gating strategy of analysis of tumor-infiltrating lymphocytes. Cell suspensions were prepared as indicated in materials and methods section. All cells were recorded, doublets and dead cells were excluded and $Cd3^{+}$ T cells were analyzed for surface expression of inhibitory T cell markers Lag-3, Ctla-4 and Pd-1.

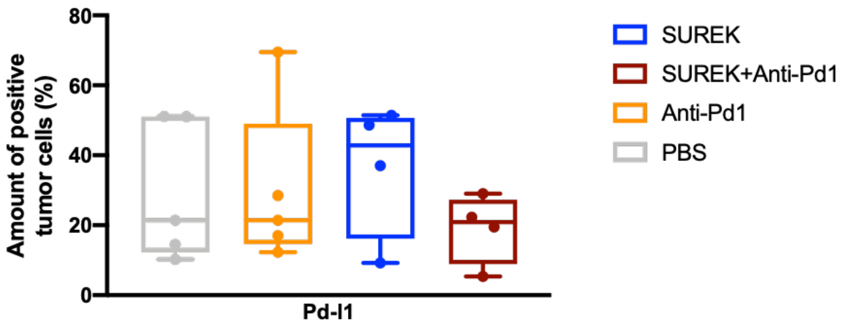

Supplementary Figure 5. Expression level of Pd-l1 on tumor cells that grew out after vaccination treatment. Tumors that grew out in mice that had been treated as indicated in Figure 4A were isolated and flow cytometrically analyzed for Pd-l1 surface expression.

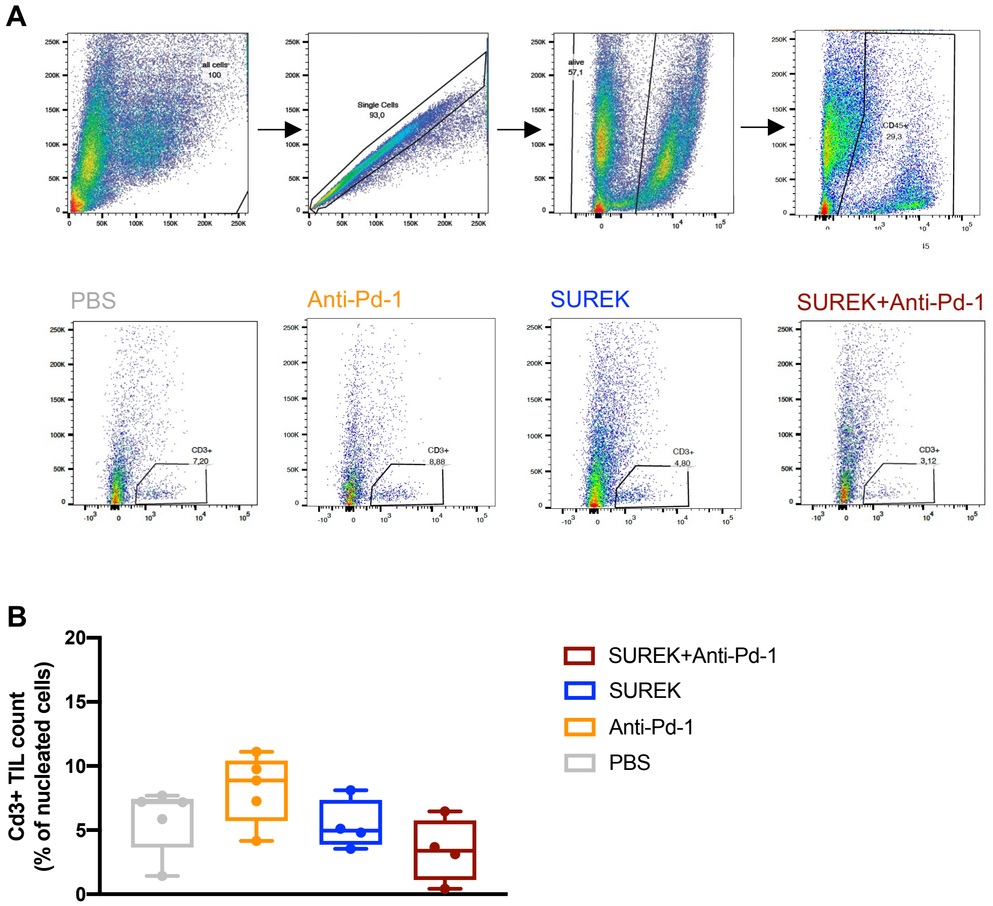

Supplementary Figure 6. Cd3^{+} TIL infiltration is reduced due to immune escape mechanisms of tumor in mice. (A) Representative dot plots show amount of Cd3^{+} tumor-infiltrating T cells (TILs) that can be found in tumors that grew out in mice that had been treated as indicated in Figure 4A. **(B)** Combined data of TILs.
